# Ratio Dependent Synergism in Azo-dye Carcinogenesis

**DOI:** 10.1038/bjc.1961.36

**Published:** 1961-06

**Authors:** J. C. Arcos, G. W. Griffith


### RATIO DEPENDENT SYNERGISM IN AZO-DYE CARCINOGENESIS*

J. C. ARCOS† AND G. W. GRIFFITH

*From the Cancer Research Laboratory, J. Hillis Miller Health Center University of Florida, Gainesville, Florida*

Received for publication April 8, 1961

A SCARCITY of information exists in the literature on the hepatocarcinogenic effects of combinations of azo dyes, similar to the competition in epithelial carcinogenesis between polycyclic hydrocarbons as described first by Lacassagne, Buu-Hoi and Rudali (1945). In the reports available on synergism among azo carcinogens only the synergistic effect of 4-dimethylaminoazobenzene (DAB) and 4′-methyl-DAB has been confirmed (MacDonald *et al.*, 1952; Corre-Hurst *et al.*, 1953). The final assessment of the synergism between DAB and 3′-methyl-DAB (3′-Me-DAB) described in the 1953 report will require further experiments, however, in view of the fact that the authors found a higher tumor incidence with DAB than with 3′-Me-DAB, which is the inverse of the generally accepted relative carcinogenicites of the two compounds. Inhibitory effect among azo carcinogens has been reported by Crabtree (1955) who described inhibition of DAB induced hepatic tumorigenesis by certain non-carcinogenic derivatives of azobenzene.

These observations have now been extended by studying the carcinogenic effect of dietary mixtures of the potent hepatic carcinogen, 3′-Me-DAB, of its relatively inactive isomer, 2-Me-DAB, and of the parent compound DAB. This report describes the synergistic effect of 3′-Me-DAB and of 2-Me-DAB when these dyes are present in a 1 : 2 proportion in the diet. However, when these two azo compounds are present in a ratio of 1 : 1, the synergistic effect is abolished, and the comparison of the tumor incidences may be considered suggestive of a limited inhibitory effect of 2-Me-DAB on 3′-Me-DAB tumorigenesis. In relation to this biological effect the phase diagram of 2-Me and 3′-Me-DAB has been studied to bring evidence for their actual similarity of spatial configuration.

### MATERIALS AND METHODS

*Care and feeding of animals.*—Sprague-Dawley male rats, weighing 180 to 230 g. at the beginning of the experiment and housed two to a cage, were used. The rats were fed a semi-synthetic diet, called hereafter basal diet, similar to the diet No. 2 used by Miller, Miller and Finger (1957). In the present diet, however, the previous salt mixture (Miller, MacDonald and Miller, 1955) has been substituted by the commercially available Salt Mixture W (Nutritional Biochemicals Corporation, Cleveland 28, Ohio). In addition to this, the diet was now supplemented

* Presented at the Fifty-first Annual Meeting of the American Association for Cancer Research, Inc., Chicago, Illinois, April 8–10, 1960 (*Proc. Am. Assoc. Cancer Res.*, 3, 92, 1960).

† Present address: U.S. Public Health Service Hospital, Research Laboratory, 210 State Street and Departments of Medicine and Biochemistry, Tulane University Medical School, New Orleans, Louisiana.

with 1·6 mg./kg. of Menadione (2-methyl-1,4-naphthoquinone) and also with 14 mg./kg. of zinc sulfate heptahydrate since the Salt mixture W does not contain zinc, which is essential to the development of the rat (Farris and Griffith, 1949). The azo compounds, DAB, 3′-Me-DAB and 2-Me-DAB were incorporated in the basal diet at the levels stated in Table I. The rats were fed basal diet for at least

TABLE I.—*Hepatic Tumour Incidence with Combinations of 3′-Me-DAB, 2-Me-DAB and DAB*

| | | | Incidence of liver tumors* (months of continuous feeding) | | | | | |
|---|---|---|---|---|---|---|---|---|
| Group | Diet | Number of rats started | 3½ | 4 | 4½ | 6 | 7 | 8 |
| A | 0·02 per cent 3′-Me-DAB + 0·04 per cent 2-Me-DAB | 23 | — | — | 0/20 | — | 5/20† | — |
| B | 0·02 per cent 3′-Me-DAB | 23 | — | — | 0/23 | — | 1/23 | — |
| C | 0·04 per cent 2-Me-DAB | 23 | — | — | 0/23 | — | 0/22 | — |
| D | 0·035 per cent 3′-Me-DAB + 0·035 per cent 2-Me-DAB | 24 | 1/24‡ | — | 10/24 | 21/23 | — | — |
| E | 0·035 per cent 3′-Me-DAB | 24 | 0/24 | — | 14/24 | 24/24 | — | — |
| F | 0·06 per cent 2-Me-DAB 3 mo. → 0·054 per cent DAB | 20 | — | 1/18 | — | — | — | 4/17 |
| G | Basal diet 3 mo. → 0·054 per cent DAB | 20 | — | 0/20 | — | — | — | 3/18 |

* Number of rats with tumors/number of rats alive.
† In addition to these five tumor bearing animals, a sixth had a teratoma of a kidney.
‡ Hepatic tumors failed to develop in an additional group of 19 rats maintained on this diet for 3½ months. This would bring the overall tumor incidence to 1/43.

1 week prior to the feeding of the dye containing diet. All diets and water were *ad libitum*. For the intermittent examinations for liver tumors exploratory laparotomy was performed under ether anesthesia. At the end of the respective experiments all survivors were sacrificed for final tumor count and extensive autopsy. All macroscopically visible nodules found during exploratory laparotomy or at the termination of experiments were examined histopathologically. When no gross tumor was apparent and no small tumor foci were visible, the liver was described as " normal " regardless of the well known cellular changes preceding the emergence of tumors, which was the criterion used by Crabtree (1955).

*Preparation of compounds and determination of phase diagrams.*—DAB was obtained commercially. 3′-me-DAB and 2-Me-DAB were prepared in our laboratory following the standardized procedure of Miller, Sapp and Miller (1949). 4-Hydroxyazobenzene was obtained in a similar way by coupling benzenediazonium-chloride to phenol in 70 per cent aqueous ethanol in the presence of 1·5 mole NaOH for each mole of phenol. At the end of coupling, the reaction mixture was brought to pH 7 and the precipitation of the dye enhanced by adding a saturated solution of sodium acetate.

Once crystallized samples were used for feeding, and three times crystallized and carefully dried samples were used for the determination of the phase diagrams. Initial thawing points and final melting points were taken in triplicate with a capillary arrangement in a Thiele tube fitted with a standardized thermometer and a magnifying lens. All melting points are corrected. The variations of the final melting points were within ±0·5 per cent.

### RESULTS AND DISCUSSION

### *Animal experiments*

The tumor incidences given for Groups A, B and C of Table I show marked synergism when 0·04 per cent 2-Me-DAB and 0·02 per cent 3′-Me-DAB are present in mixture in the diet fed for 7 months. That the observed effect corresponds to true synergism is indicated by the fact that in Group A the two compounds together (at the concentrations as they are present singly in Groups B and C) produced 5 tumor bearers in 20 rats (25 per cent incidence), compared with 1 tumor bearer produced by these dyes administered separately to a total of 45 rats in Groups B and C (2·2 per cent). This difference is statistically significant, since $P < 0{\cdot}01$ using Fisher's exact test for $2 \times 2$ contingency tables. On the other hand, a comparison of Groups C, D and E indicates that when these two dyes are present in the diet in equal proportion, no synergism is observed although the level of 2-Me-DAB in the test Group D (0·035 per cent) is similar to that in test Group A (0·04 per cent). For the difference between the tumor incidences in Groups C plus E, and Group D, at $4\frac{1}{2}$ months $\chi^2 = 0{\cdot}545$ was found, corresponding to $P \simeq 0{\cdot}45$ on 1 degree of freedom.

Two alternative assumptions may be made for the absence of synergism in the latter experiment : (1) If the tumor incidence values in these various groups are plotted against the respective time intervals, it may be seen that the slope of the curve of Group E is much greater than the one of the synergistic Group A. For this reason the absence of synergism in Group D may be interpreted as resulting from a " covering " of the synergistic effect of 0·035 per cent 2-Me-DAB by the rapidity of tumour development due to the presence of 3′-Me-DAB at the 0·035 per cent level. (2) When both 2-Me and 3′-Me-DAB are fed at identical levels of 0·035 per cent each (Group D), competitive antagonism may occur at certain cellular binding sites compensating the synergistic effect of 2-Me-DAB. This possibility of competitive antagonism is also suggested by : (*a*) the presence of both compounds in all liver cell fractions of treated rats (Price *et al.*, 1949, 1950 ; Hultin, 1956) ; (*b*) the close similarity of the spatial configurations of the two dyes (see " Phase Diagrams "), and (*c*) the fact that both 2-Me and 3′-Me-DAB are linked in the soluble cytoplasm to the same electrophoretic protein fraction (Sorof *et al.*, 1951 ; Wirtz and Arcos, 1958). The comparison of the tumor incidences at $4\frac{1}{2}$ months in Group D (10/24) and Group E (14/24) may actually be considered as suggestive of a limited inhibition of 3′-Me-DAB tumorigenesis by 2-Me-DAB. The statistical significance of this apparent difference is, however, low ($P \simeq 0{\cdot}30$). Unfortunately, due to the difficulties in carrying out large numbers of laparotomies within short intervals of time and because these experiments had to be terminated, a more solid assessment of the latter data could not be obtained.

In another experiment, feeding of 2-Me-DAB preceeded feeding the medium active DAB (Groups F and G). The rats of the test Group F were fed 0·06 per cent 2-Me-DAB for 3 months, which has been shown (Miller and Miller, 1947) to be the time when maximum level of bound dye is reached with this compound under similar dietary conditions. After this period, feeding was immediately continued with 0·054 per cent DAB. The control Group G was fed basal diet for 3 months and then fed the same level of DAB. No difference could be observed between the tumor incidences in these groups ($P > 0{\cdot}90$). Thus, previous saturation of cellular binding sites with 2-Me-DAB does not affect DAB induced tumorigenesis.

FIG. 1.

FIG. 2.

FIG. 3.

Fig. 4.

Fig. 5.

Fig. 1 to 5.—Phase diagrams of binary mixtures of azo dyes showing polymorphic transformations and regions of continuous miscibility. Final melting points (solid line) varied within $\pm 0{\cdot}5$ per cent. The dotted line curves give the initial thawing points. The figures correspond to the following pairs of compounds : Fig. 1, 3′-Me-DAB plus 2-Me-DAB ; Fig. 2, DAB plus 4-hydroxyazobenzene ; Fig. 3, 3′-Me-DAB plus 4-hydroxyazobenzene ; Fig. 4, DAB plus 2-Me-DAB ; Fig. 5, 3′-Me-DAB plus 20-methylcholanthrene.

Histologically the tumors were classified mainly as hepatomas and cholangiocarcinomas and these two histopathological types were observed in about equal proportion. Three of the 5 tumors found in Group A fed 0·04 per cent 2-Me-DAB plus 0·02 per cent 3′-Me-DAB were, however, cholangiomas.

The cellular mechanism that may account for the observed synergistic effect must involve more than one receptor site (*cf.* Ariens, van Rossum and Simonis, 1956 ; Veldstra, 1956) and this is consistent with the general distribution of bound, aminoazo dyes among all cell components of the liver (Price *et al.*, 1949, 1950 ; Hultin, 1956). The liver enzyme systems which metabolize various drugs and inactivate carcinogenic azo dyes by *N*-demethylation, azo double bond reduction and ring hydroxylation (*e.g.*, Mueller and Miller, 1949 ; Conney *et al.*, 1957 ; Conney, Miller and Miller, 1957 ; Conney *et al.*, 1959) have been identified in the " microsome " fraction. These enzyme systems are altered and functionally eliminated at high levels of feeding 3'-Me-DAB (Miller, Miller and Brown, 1952) at a rate which may be expected to be linear with the concentration of the dye fed. An approximate proportionality is in fact shown by the carcinogenicities of 0·06 per cent and 0·035 per cent 3'-Me-DAB diets : 100 per cent tumour incidence has been observed in a group of 16 rats after 4 months of continuous feeding 0·06 per cent 3'-Me-DAB, while Table I indicates that with 0·035 per cent diet only 14 tumour bearing rats out of 24 have been found after $4\frac{1}{2}$ months (Group E). This approximate linearity of the dose-response fails, however, when the responses to the 0·035 and 0·02 per cent diets are compared, since no tumor bearing animal is found when the latter diet is fed for $4\frac{1}{2}$ months to 23 rats (Group B). The relative inactivity of the 0·02 per cent 3'-Me-DAB diet may be due to a rate of metabolic inactivation which balances the rate of arrival of the dye in the liver. Consequently, from the standpoint of the process of carcinogenesis, the microsomal enzyme systems inactivating 3'-Me-DAB may be considered " sites of loss " in the sense described by Veldstra (1956).

Carcinogenesis may result then from : (*a*) intake of 3'-Me-DAB in excess over the metabolic capacity of the enzyme systems in the microsomes, which excess will be available for alteration of this cell organelle proper (Arcos and Arcos, 1958 ; Porter and Bruni, 1959), and of other cellular components, *e.g.* the mitochondria (Arcos, Griffith and Cunningham, 1960), at a rate which is proportional to the amount of non-metabolized dye, such as may be the case with the 0·035 and 0·06 per cent diets, or (*b*) competition of 2-Me and 3'-Me-DAB at " sites of loss " (*cf.* Veldstra, 1956) making 3'-Me-DAB available at other cellular sites the alterations of which are involved in the succession of steps leading to carcinogenesis (Arcos, Gosch and Zickafoose, 1961).

*Phase diagrams*

Synergism and antagonism as the response to simultaneously present biologically active compounds often involves close steric molecular similarity (*e.g.* Veldstra, 1956). Because of steric hindrance between the 2-methyl group and the α-azo nitrogen in 2-Me-DAB (Arcos and Arcos, 1958), it may be expected that the spatial configuration of this compound and of 3'-Me-DAB may not be so closely similar as suggested by the simple observation of the structural formulae. Steric hindrance in 2-Me-DAB is also suggested by the high basicity of the amino nitrogen (Cilento, 1960), because of interference with the resonance involving the azo double bond and consequent increase of the electron density at the amino nitrogen. For these reasons, the phase diagram of these dyes, and comparatively of binary mixtures of other azo compounds have been studied since it is well known that polymorphic transformations or continuous mixed crystal formation, that may be detected in such diagrams, indicate interchangeability in mixed crystal lattices (*e.g.* Eitel, 1945) which in turn is dependent on close steric similarity or complementarity (*e.g.* Timmermans, 1939 ; Fredga, 1945).

The phase diagrams obtained with mixtures of various azo dyes suggest that the interchangeability of 3'-Me-DAB and 2-Me-DAB in their mixed crystal lattices is relatively higher than in mixed crystals of certain structurally related azo dyes. In fact, the phase diagram of these two compounds (Fig. 1) indicates the existence of at least four regions of polymorphic transformations, in addition to a rather flat eutectic region around 90 per cent 2-Me-DAB. Moreover, the flatness of this region and the even concavity of the thawing point curve (dotted line) relatively to the final melting point curve (solid line) is suggestive, that other polymorphic transformations might have remained undetected and so that the two compounds possibly form a nearly continuous series of solid solutions. The temperature differences involved in the regions of polymorphic transformations are significant because of the narrow range of the final melting points ($\pm$0·5 per cent). Thus, there is no overlapping between the minima representing these polymorphic transformations and the points (with the respective $\pm$0·5 per cent ranges) corresponding to them on a curve which would join the remaining points of the final melting point curves. Decreasing numbers of polymorphic transformations may be observed in the phase diagrams of other azo dyes (Fig. 2 to 5). However, the curve of the thawing points in Fig. 4 is suggestive that, also with DAB plus 2-Me-DAB, other regions of polymorphic transformations may exist between 40 and 100 per cent DAB. The anomaly in Fig. 2 and 3 that sharp eutectic points coexist with rather flat shoulders up to 60–70 per cent DAB or 3′-Me′DAB, may be due to more stable molecular combinations by interaction between the phenolic hydroxyl in 4-hydroxyazobenzene and the basic nitrogen in the two aminoazo dyes. In the phase diagram of 3′-Me-DAB plus 20-methylcholanthrene (Fig. 5) a very noticeable polymorphic transformation has been observed at 10 per cent 3′-Me-DAB, in addition to an expected sharp eutectic point.

The above phase diagrams recall the interesting work of Müller, Görlich and Kahofer (1954) who observed a correlation between mixed crystal formation in binary mixtures of polycyclic hydrocarbons and antagonism of these compounds in epithelial carcinogenesis. Applying the well known concept that for heterogenous organic catalysis a correlation of molecular geometry must exist between the catalytic site and the organic substrate, these authors suggested that the mixed crystal formation of polycyclic hydrocarbons may imply interchangeability at cellular receptor sites.

### SUMMARY

1. There is synergism in hepatic tumor induction when a diet containing 0·04 per cent 2-Me-DAB plus 0·02 per cent 3′-Me-DAB is fed to rats. This synergism is abolished by raising the concentration of 3′-Me-DAB to 0·035 per cent. Prolonged previous feeding with 0·06 per cent 2-Me-DAB does not affect the tumor incidence produced by a 0·054 per cent DAB diet.

2. Regions of polymorphic transformations that may be observed in the phase diagram of 2-Me-DAB plus 3′-Me-DAB suggest that, in spite of the steric hindrance present in the former compound, there is a close similarity in their spatial configurations. For comparison, phase diagrams of pairs of other azo dyes have been determined.

The authors are greatly indebted to Dr. Steven P. H. Mandel for the statistical calculations and related evaluation of the results, to Dr. Mary F. Argus for discussion and criticism of the manuscript, to Dr. Cornelia Hoch-Ligeti and Dr. Joseph Simon for the histopathological examinations, and to Dr. Francis E. Ray for support during the early stages of these investigations.

This work was supported in part by the U.S. Public Health Service Research Grant C-4351.

### REFERENCES

ARCOS, J. C. AND ARCOS, M.—(1958) *Biochim. biophys. Acta*, **28**, 9.—(1958) *Arzneimittelforschung*, **8**, 486.
*Idem*, GOSCH, H. H. AND ZICKAFOOSE, D.—(1961) *J. biophys. biochem. Cytol.*, in press.
*Idem*, Griffith, G. W., and Cunningham, R. W.—(1960) *Ibid.*, **7**, 49.
ARIENS, E. J., VAN ROSSUM, J. M. AND SIMONIS, A. M.—(1956) *Arzneimittelforschung*, **6**, 737.
CILENTO, G.—(1960) *Cancer Res.*, **20**, 120.
CONNEY, A. H., BROWN, R. R., MILLER, J. A. AND MILLER, E. C.—(1957) *Ibid.*, **17**, 628.
*Idem*, GILLETTE, J. R., INSCOE, J. K., TRAMS, E. R. AND POSNER, H. S.—(1959) *Science*, **130**, 1478.
*Idem*, MILLER, E. C. AND MILLER, J. A.—(1957) *J. biol. Chem.*, **228**, 753.
CORRE-HURST, L., BUU-HOI, N. P., ROYER, R., AND BIZZINI, B.—(1953) *Bull. Ass. franç. Cancer*, **40**, 397.
CRABTREE, H. G.—(1955) *Brit. J. Cancer*, **11**, 310.
EITEL, H.—(1945) " Die Heterogenen Schmelzgleichgewichte Silikatischer Mehrstoff-Systeme." Leipzig (Barth), pp. 9–12.
FARRIS, E. J. AND GRIFFITH, J. Q.—(1949) ' The Rat in Laboratory Investigation.' Philadelphia (Lippincott), pp. 80, 98.
FREDGA, A.—(1945) ' On the Use of Melting Point Curves for the Establishment of Steric Relationships between Optically Active Compounds,' in Tiselius and Pedersen, Ed., ' The Svedberg 1884–1944.' Uppsala (Almqvist & Wiksells), pp. 261–273.
HULTIN, T.—(1956) *Exp. Cell Res.*, **10**, 71, 697.
LACASSAGNE, A., BUU-HOI, N. P. AND RUDALI, G.—(1945) *Brit. J. exp. Path.*, **26**, 5.
MACDONALD, J. C., MILLER, E. C., MILLER, J. A. AND RUSCH, H. P.—(1952) *Cancer Res.*, **12**, 50.
MILLER, E. C., MACDONALD, J. C. AND MILLER, J. A.—(1955) *Ibid.*, **15**, 320.
*Idem* and Miller, J. A.—(1947) *Ibid.*, **7**, 468.
*Idem*, MILLER, J. A. AND BROWN, R. R.—(1952) *Ibid.*, **12**, 282.
MILLER, J. A., MILLER, E. C. AND FINGER, G. C.—(1957) *Ibid.*, **17**, 387.
*Idem*, SAPP, R. W. AND MILLER, E. C.—(1949) *Ibid.*, **9**, 652.
MÜLLER, A., GÖRLICH, P. AND KAHOFER, L.—(1954) *Mh. Chem.*, **85**, 906.
MUELLER, G. C. AND MILLER, J. A.—(1949) *J. biol. Chem.*, **180**, 1125.
PORTER, K. R. AND BRUNI, C.—(1959) *Cancer Res.*, **19**, 997.
PRICE, J. M., MILLER, E. C., MILLER, J. A. AND WEBER, G. M.—(1949) *Ibid.*, **9**, 398.—(1950) *Ibid.*, **10**, 18.
SOROF, S., COHEN, P. P., MILLER, E. C. AND MILLER, J. A.—(1951) *Ibid.*, **11**, 383.
TIMMERMANS, J.—(1939 *Bull. Soc. chim. Belges*, **48**, 33.
VELDSTRA, H.—(1956) *Pharmacol. Rev.*, **8**, 339.
WIRTZ, G. H. AND ARCOS, J. C.—(1958) *Experientia*, **14**, 177.


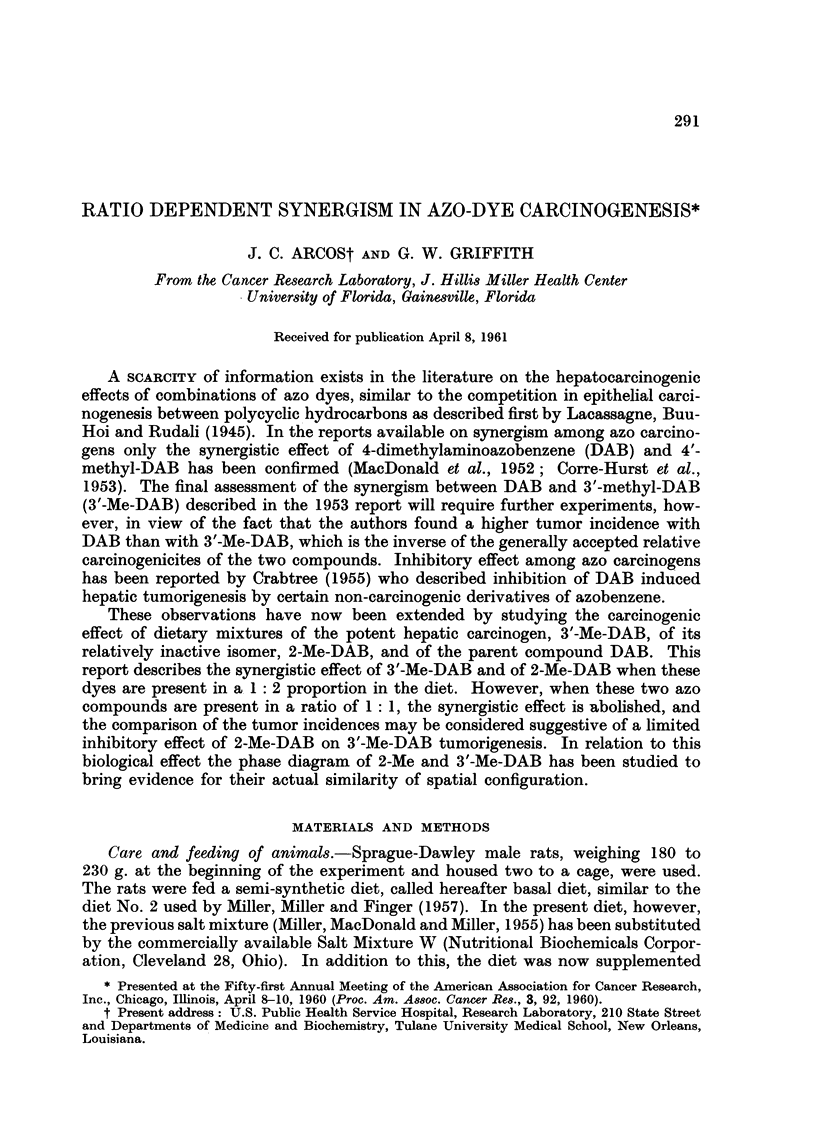

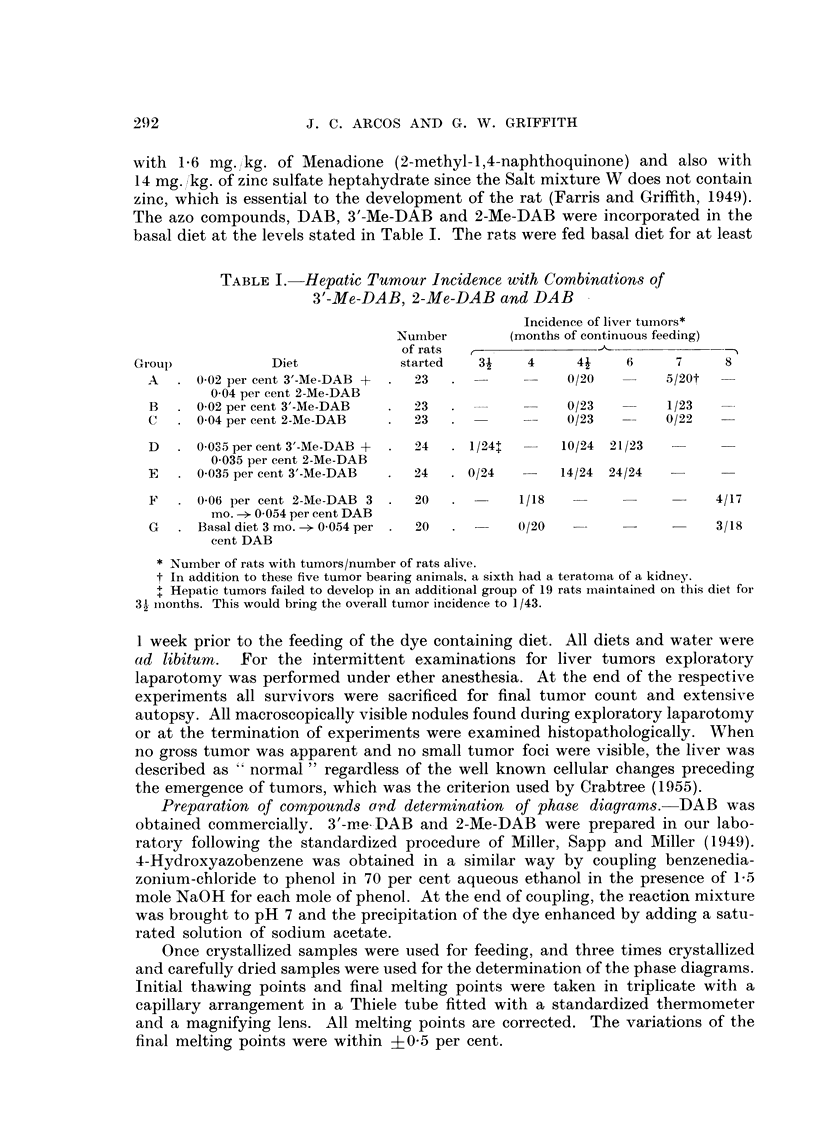

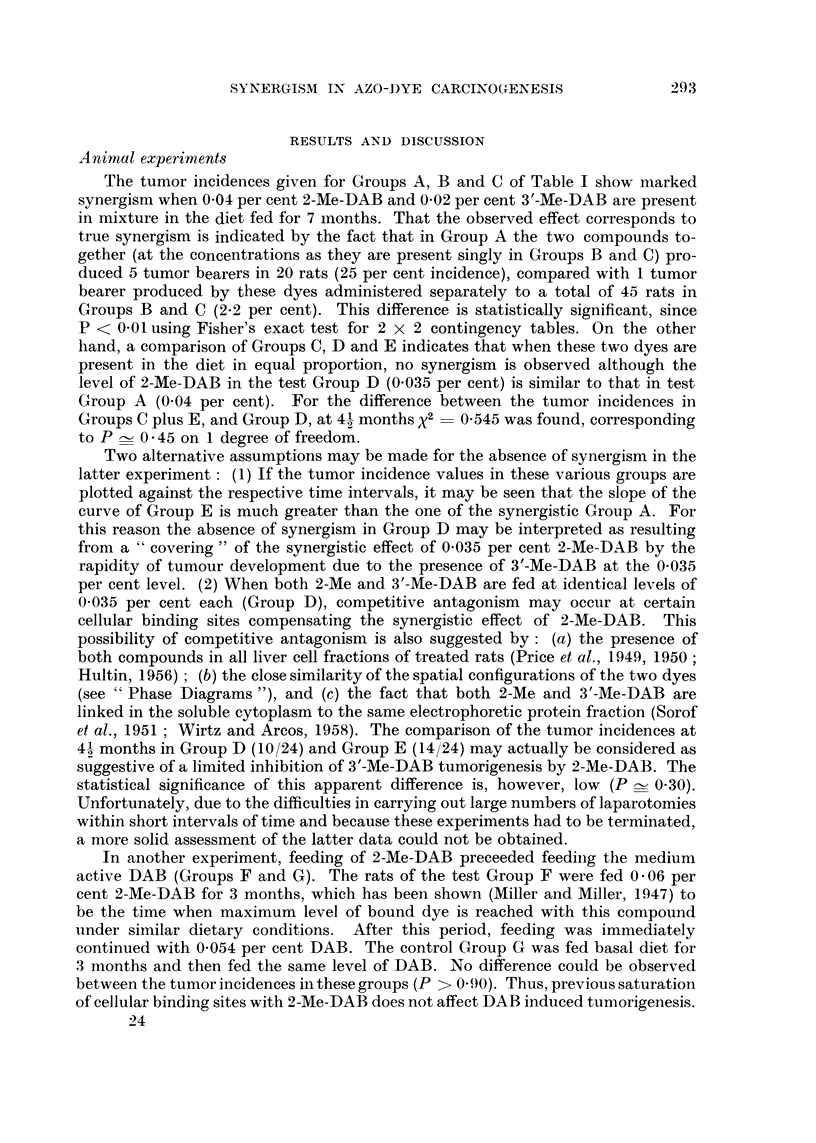

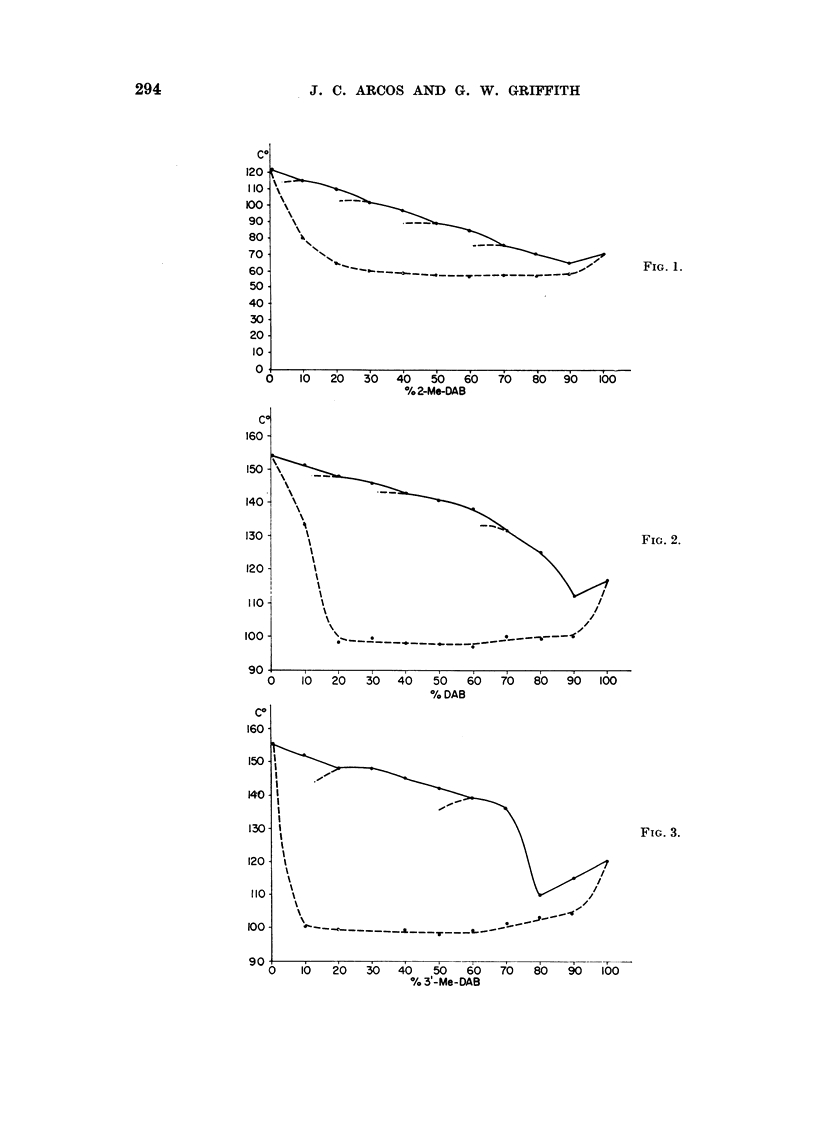

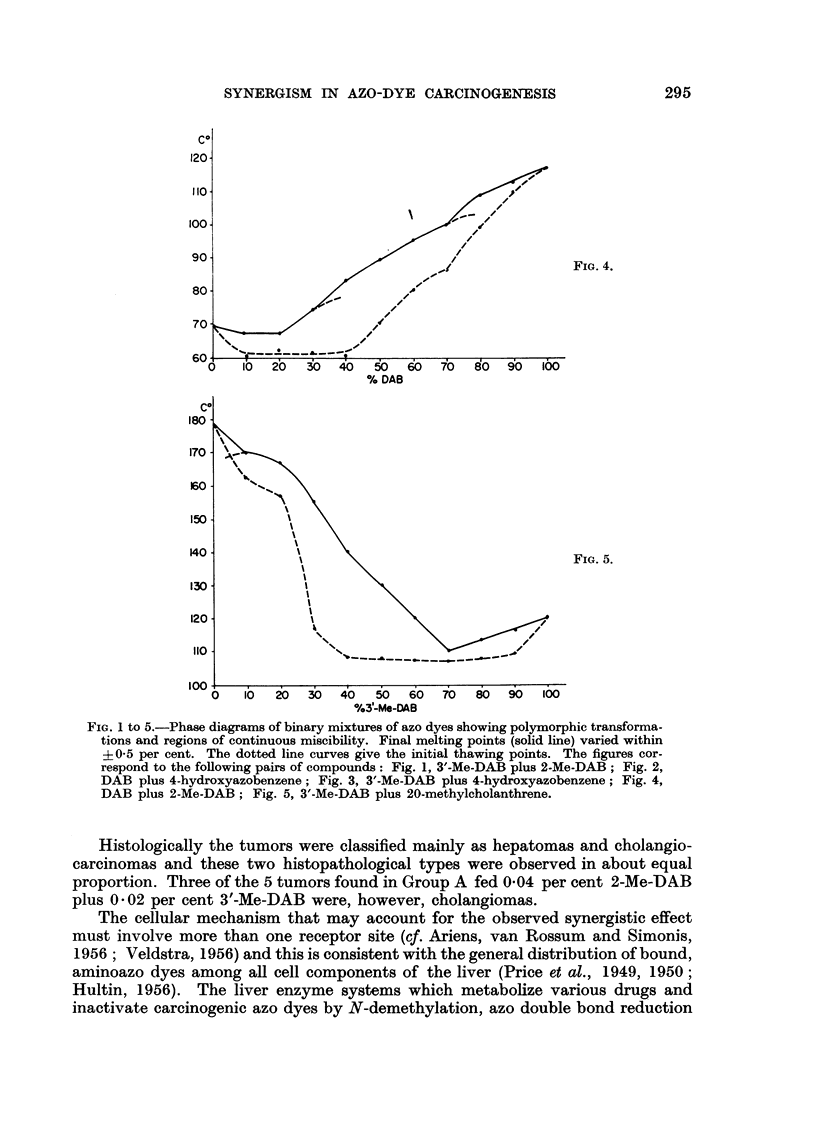

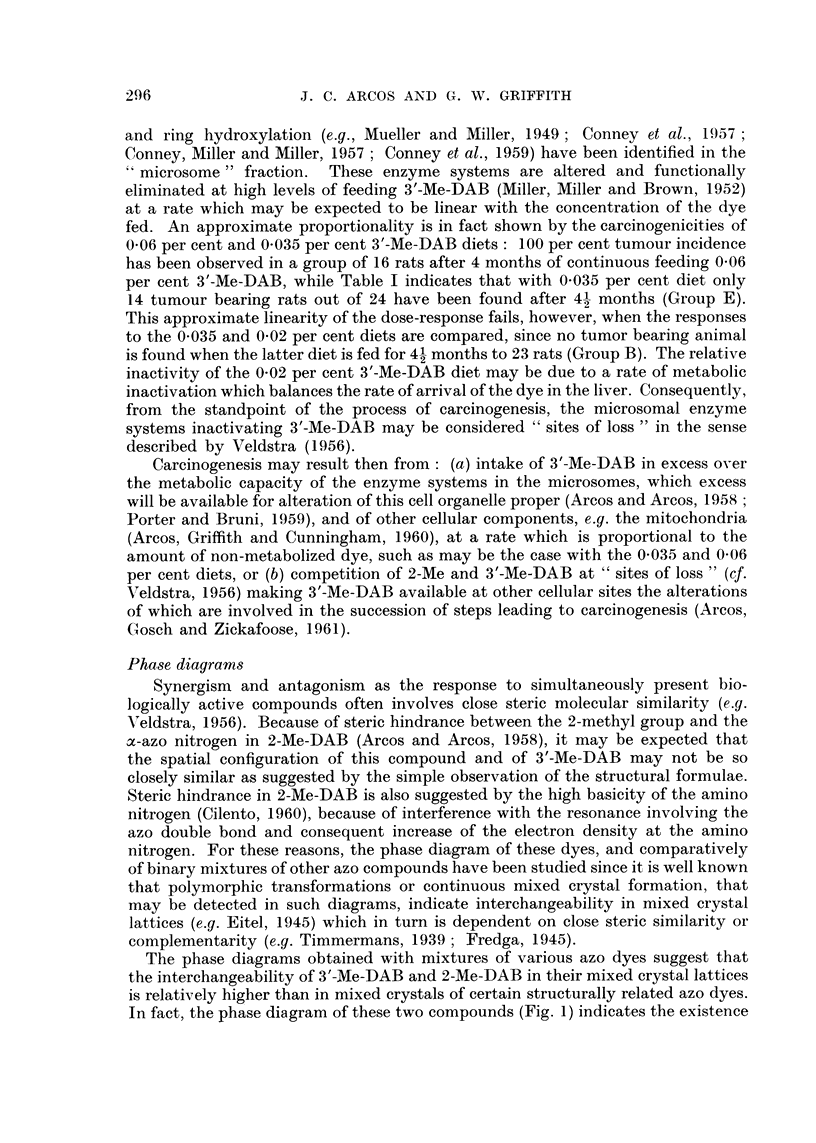

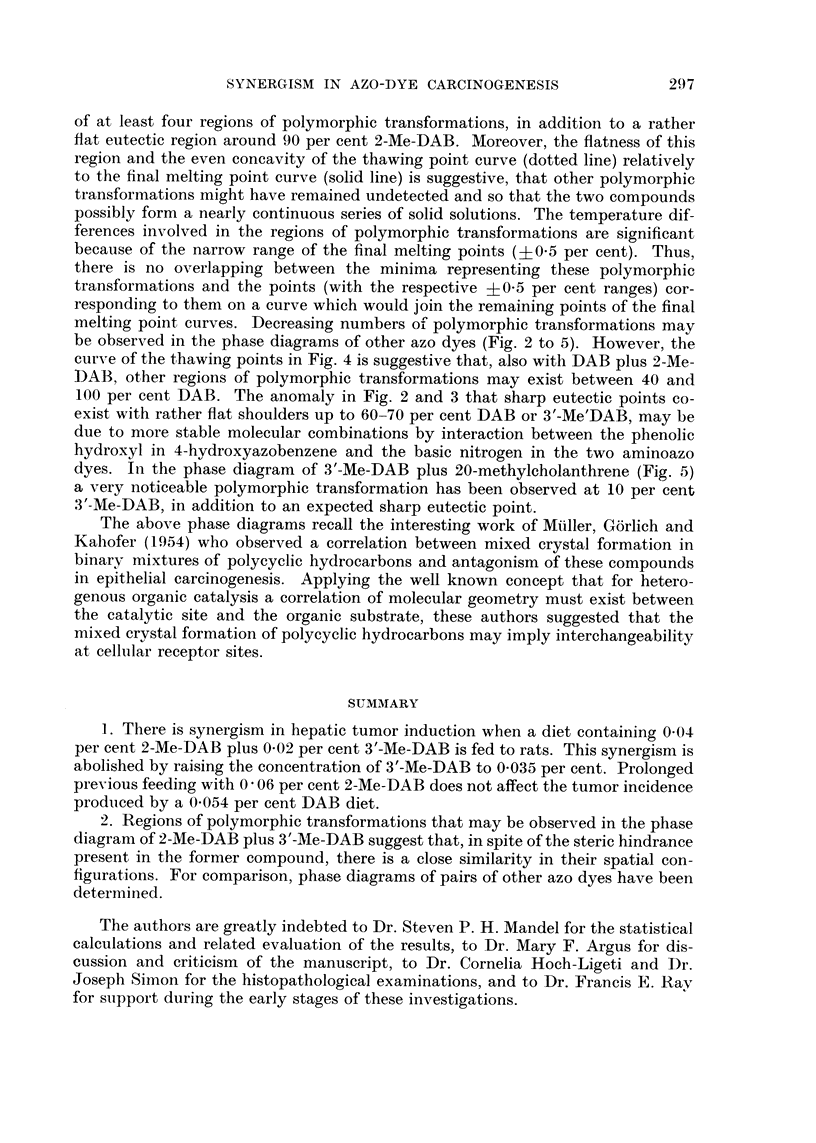

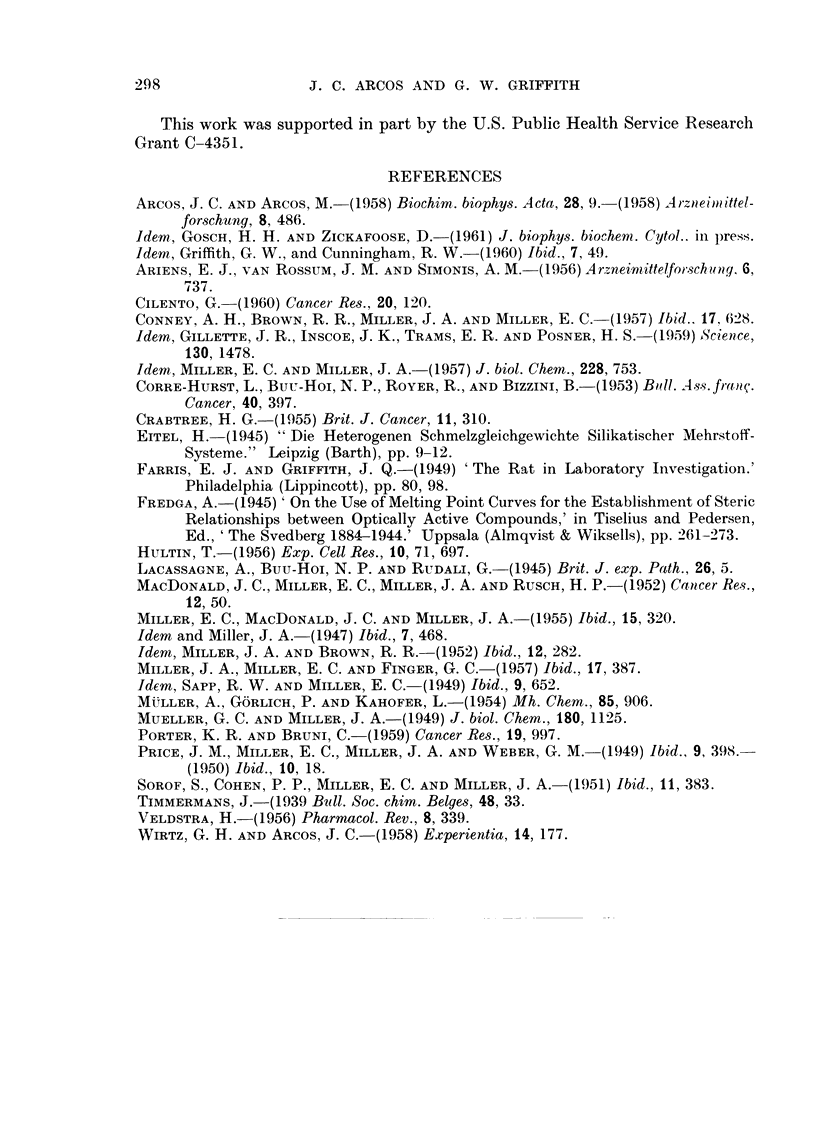


## References

[OCR_00435] ACROS M., ARCOS J. C. (1958). A correlation of constitution and carcinogenic activity. I. A study of the essential molecular parameters.. Arzneimittelforschung.

[OCR_00449] CONNEY A. H., GILLETTE J. R., INSCOE J. K., TRAMS E. R., POSNER H. S. (1959). Induced synthesis of liver microsomal enzymes which metabolize foreign compounds.. Science.

[OCR_00453] CONNEY A. H., MILLER E. C., MILLER J. A. (1957). Substrate-induced synthesis and other properties of benzpyrene hydroxylase in rat liver.. J Biol Chem.

[OCR_00455] CORRE-HURST L., BUU-HOI N. P., ROYER R., BIZZINI B. (1953). Compétitions et synergies dans la production de cancers du foie chez le rat par des azoïques de constitution chimique voisine de celle du jaune de beurre.. Bull Assoc Fr Etud Cancer.

[OCR_00473] HULTIN T. (1956). The distribution of protein-bound azo dye in subfractions of liver cytoplasm fractions.. Exp Cell Res.

[OCR_00481] MILLER E. C., MACDONALD J. C., MILLER J. A. (1955). Inability of 4-dimethylamino azobenzene to act as a major source of labile methyl groups.. Cancer Res.

[OCR_00487] MILLER J. A., SAPP R. W., MILLER E. C. (1949). The carcinogenic activities of certain halogen derivatives of 4-dimethylaminoazobenzene in the rat.. Cancer Res.

[OCR_00491] PORTER K. R., BRUNI C. (1959). An electron microscope study of the early effects of 3'-Me-DAB on rat liver cells.. Cancer Res.

[OCR_00497] SOROF S., COHEN P. P., MILLER E. C., MILLER J. A. (1951). Electrophoretic studies on the soluble proteins from livers of rats fed aminoazo dyes.. Cancer Res.

[OCR_00499] VELDSTRA H. (1956). Synergism and potentiation with special reference to the combination of structural analogues.. Pharmacol Rev.

[OCR_00501] WIRTZ G. H., ARCOS J. C. (1958). Studies on the dye-binding fraction of soluble liver proteins from rats fed aminoazo dyes.. Experientia.

